# *ATR* is a MYB regulated gene and potential therapeutic target in adenoid cystic carcinoma

**DOI:** 10.1038/s41389-020-0194-3

**Published:** 2020-01-30

**Authors:** Mattias K. Andersson, Giovanna Mangiapane, Paloma Tejera Nevado, Alexia Tsakaneli, Therese Carlsson, Gabriele Corda, Valentina Nieddu, Carla Abrahamian, Olesya Chayka, Lilam Rai, Michael Wick, Amanda Kedaigle, Göran Stenman, Arturo Sala

**Affiliations:** 1 Sahlgrenska Cancer Center, Department of Pathology, University of Gothenburg, Gothenburg, Sweden; 2 Department of Life Sciences, Research Institute for the Environment, Health and Societies, Brunel University London, UB8 3PH Uxbridge, UK; 3 Sahlgrenska Cancer Center, Department of Medical Chemistry and Cell Biology, University of Gothenburg, Gothenburg, Sweden; 4 South Texas Accelerated Research Therapeutics (START), San Antonio, TX 78229 USA; 5 Adenoid Cystic Carcinoma Research Foundation, Needham, MA 02494 USA

**Keywords:** Head and neck cancer, Checkpoint signalling

## Abstract

Adenoid cystic carcinoma (ACC) is a rare cancer that preferentially occurs in the head and neck, breast, as well as in other sites. It is an aggressive cancer with high rates of recurrence and distant metastasis. Patients with advanced disease are generally incurable due to the lack of effective systemic therapies. Activation of the master transcriptional regulator MYB is the genomic hallmark of ACC. *MYB* activation occurs through chromosomal translocation, copy number gain or enhancer hijacking, and is the key driving event in the pathogenesis of ACC. However, the functional consequences of alternative mechanisms of *MYB* activation are still uncertain. Here, we show that overexpression of MYB or MYB-NFIB fusions leads to transformation of human glandular epithelial cells in vitro and results in analogous cellular and molecular consequences. MYB and MYB-NFIB expression led to increased cell proliferation and upregulation of genes involved in cell cycle control, DNA replication, and DNA repair. Notably, we identified the DNA-damage sensor kinase ATR, as a MYB downstream therapeutic target that is overexpressed in primary ACCs and ACC patient-derived xenografts (PDXs). Treatment with the clinical ATR kinase inhibitor VX-970 induced apoptosis in MYB-positive ACC cells and growth inhibition in ACC PDXs. To our knowledge, ATR is the first example of an actionable target downstream of MYB that could be further exploited for therapeutic opportunities in ACC patients. Our findings may also have implications for other types of neoplasms with activation of the *MYB* oncogene.

## Introduction

Adenoid cystic carcinoma (ACC) is a clinically challenging tumor that preferentially occurs in the salivary glands but may also arise in other exocrine glands such as those of the breast, prostate, skin, sinonasal tract, tracheobronchial tree, and female genital tract^1^. ACC can occur in all age groups but is commonly diagnosed between 50 and 60 years of age^2^. It is usually a slow growing but aggressive cancer with an often protracted clinical course and a fatal outcome. ACCs are treated with surgery and adjuvant radiotherapy. However, the majority of patients develop recurrences and/or distant metastases over time and, since there are no effective systemic therapies available for ACC, patients with advanced disease are generally incurable^3,4^. The genomic hallmark of ACC is a recurrent t(6;9)(q23;p23) translocation^5^ that results in a fusion between the *MYB* and *NFIB* genes^6^.

MYB is an oncogenic transcription factor that regulates proliferation and differentiation of in particular hematopoetic and colonic stem and progenitor cells^7^. NFIB is a transcriptional regulator that controls cell division, differentiation, and viability^8^. In the MYB-NFIB fusions, the DNA-binding and transactivation domains of MYB are fused to the C-terminal of NFIB, often encoded only by the last exon, leading to overexpression of MYB and loss of negative regulatory elements in the C-terminal part of MYB^6^. In addition to gene fusion, *MYB* may be activated by copy number gain or juxtaposition of enhancer elements from *NFIB*, *RAD51B* or *TGFBR3*^9,10^. These rearrangements result in overexpression of a normal wild-type MYB protein, whereas the fusion events commonly result in expression of truncated MYB proteins. In a subset of ACCs, *MYB* is replaced by the closely related *MYBL1* gene linked to *NFIB*, or other fusion partners, resulting in gene fusions likely to have the same oncogenic properties as the MYB fusions^11,12^. MYB activation occurs in the vast majority of ACCs and is therefore considered to be the main oncogenic driver of the disease and a potential therapeutic target^1^. MYB overexpression is also a useful diagnostic biomarker to distinguish ACC from other types of salivary gland tumors^13^. The concept that MYB is a key driver of ACC is corroborated by whole-exome sequencing and arrayCGH studies of salivary and breast ACCs showing that ACC generally has a stable genome with few other mutations and copy number alterations^9,14,15^. Moreover, recent studies have demonstrated that knockdown of *MYB-NFIB* expression in cultured, fusion-positive ACC cells results in reduced cell proliferation and decreased ACC spherogenesis under anchorage-independent growth conditions^16^.

Although there is substantial evidence indicating a key role for MYB in ACC pathogenesis, experimental evidence demonstrating that MYB can transform normal human glandular epithelial cells is lacking. Moreover, since ACC cells are exceedingly difficult to grow in culture, preclinical therapeutic target discovery downstream of MYB is severely hampered by the lack of established cell lines^16,17^. Here, we investigate the transforming potential and molecular consequences of MYB and MYB-NFIB overexpression in human mammary epithelial cells and cultured ACC cells. We identify the DNA-damage sensor kinase ATR as a MYB downstream therapeutic target that is overexpressed in ACC and show that treatment with a phase 2 ATR kinase inhibitor induce apoptosis in MYB-positive ACC cells and growth inhibition in ACC patient-derived xenografts (PDXs).

## Results

### MYB and MYB-NFIB overexpression promote proliferation of human breast epithelial cells

**Fig. 1:**
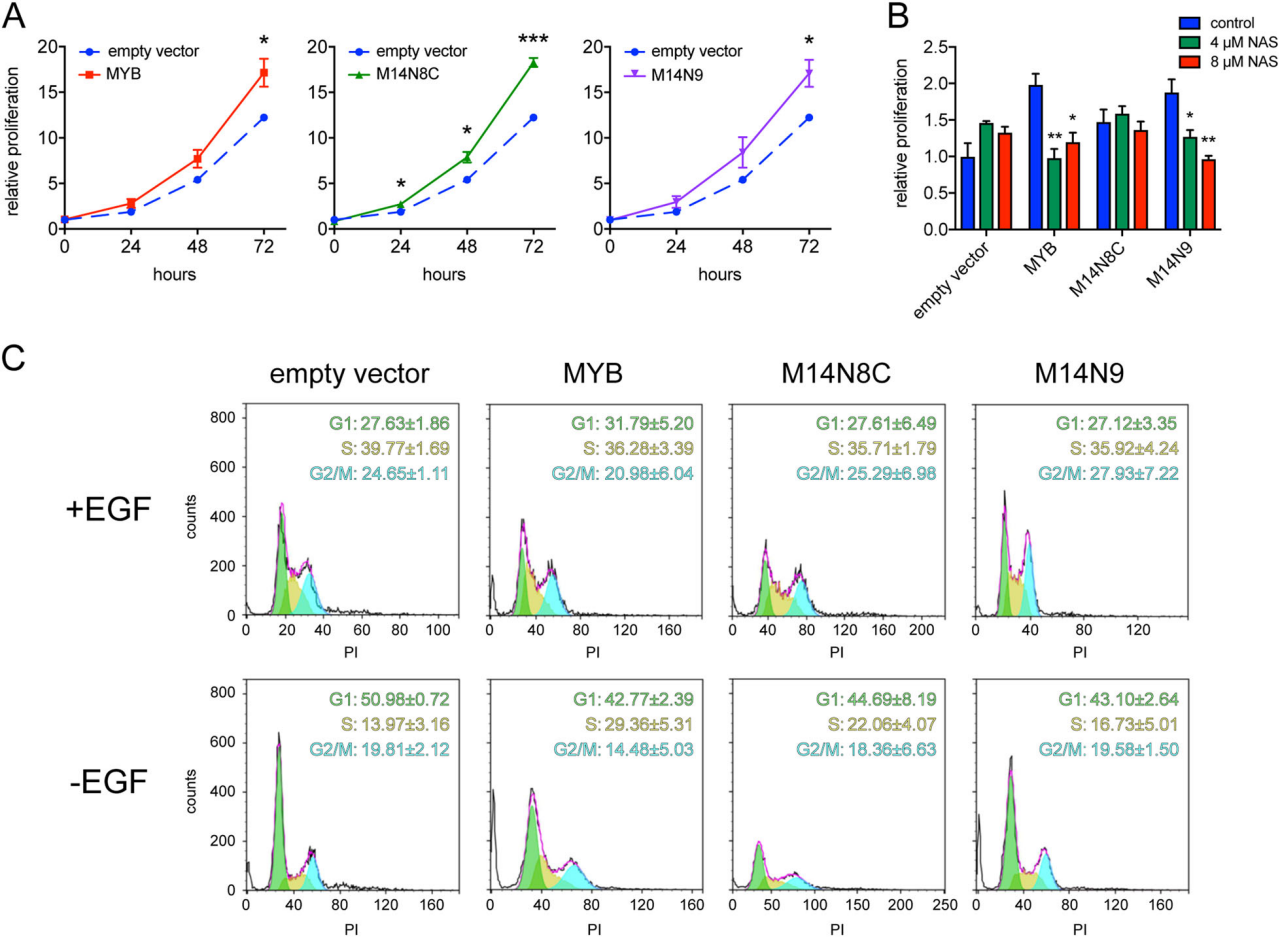
Overexpression of MYB or MYB-NFIB fusions promote growth of cultured human breast epithelial cells. **a** Analysis of proliferation of MCF10A cells transduced with retroviral expression vectors with *MYB* or two *MYB-NFIB* fusion variants (M14N8C and M14N9) using the MTT assay. Cells transduced with empty vectors served as control. Error bars indicate standard error of the mean for triplicate wells (*n* = 3). **b** MCF10A cells transduced with *MYB* or *MYB-NFIB* constructs were cultured for 48 h in the presence or absence of the MYB inhibitor Naphthol AS phosphate. Error bars indicate standard error of the mean for triplicate wells (*n* = 3). **c** Cell cycle profiles of transduced MCF10A cells in the presence or absence of EGF for 48 h. NAS – Naphthol AS phosphate, PI – propidium iodide. Asterisks indicate statistical significance with *P *<* 0.05; **P < 0.01; ***P < 0.001.

To study the transforming potential of MYB and MYB-NFIB in non-tumorigenic glandular epithelial cells, we generated stable MCF10A cell lines overexpressing wild-type *MYB* or two common variants of the *MYB-NFIB* fusion (M14N8C and M14N9). Ectopic expression of the different MYB isoforms was confirmed by immunoblot analysis (Supplementary Fig. 1). MYB and MYB-NFIB overexpressing cells showed similar levels of increased proliferation compared with cells infected with empty vectors (Fig. 1a). To study whether this effect was MYB-dependent, we treated the cells with naphthol phosphate (NAS), an inhibitor of the interaction of MYB and CREB, with the kix-domain of the CBP co-activator^18,19^. NAS treatment reduced proliferation of MYB and MYB-NFIB overexpressing cells whereas it did not significantly affect the control cells (Fig. 1b). This indicates that the increased proliferation is driven by MYB or MYB-NFIB overexpression and is not a consequence of clonal selection of the transduced cells.

### MYB and MYB-NFIB promote EGF-independent cell cycle progression and three-dimensional growth of human breast epithelial cells

**Fig. 2:**
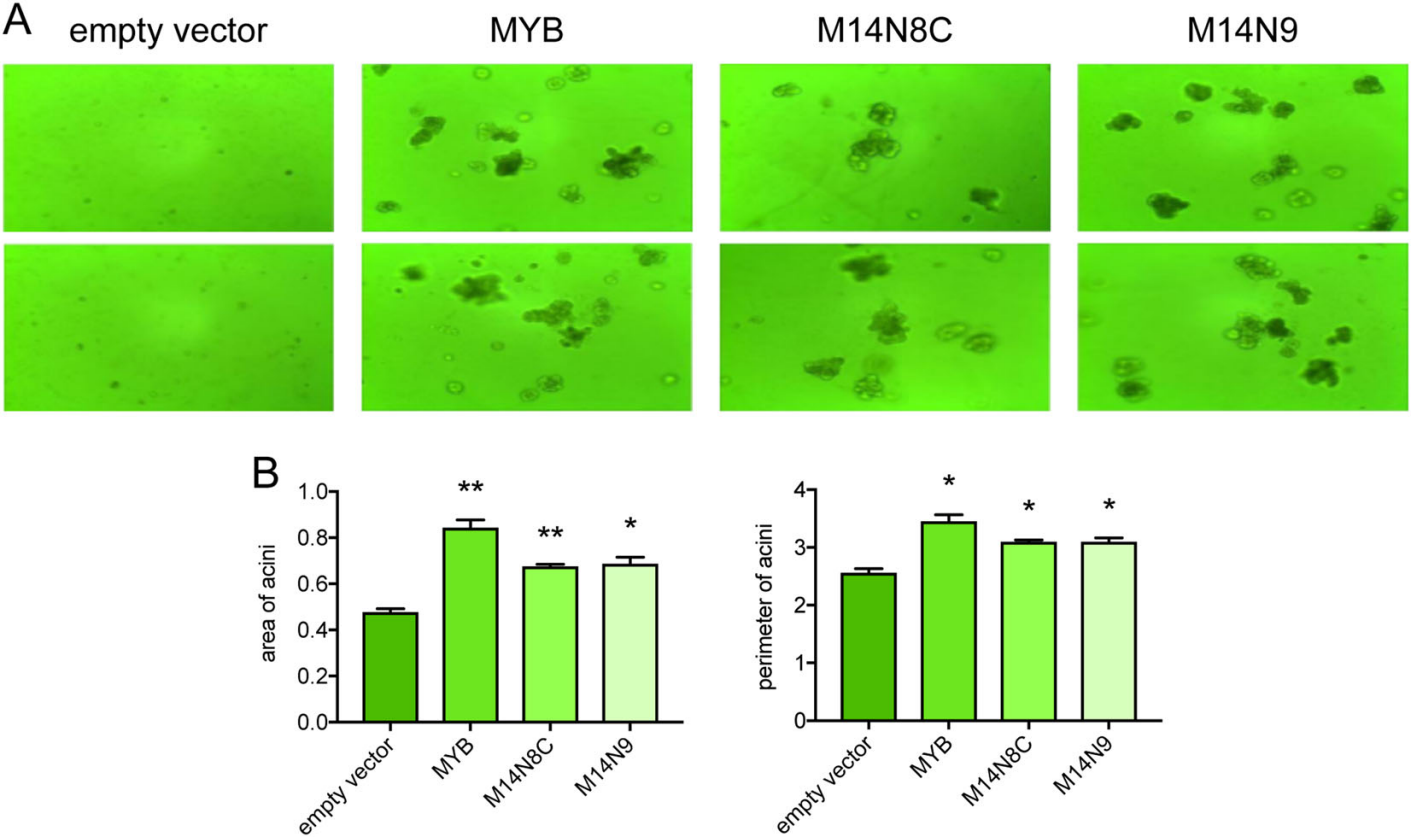
MYB and MYB-NFIB promote growth of human breast epithelial cells as organoids in three-dimensional culture. **a** Organoids formed by MYB or MYB-NFIB fusion (M14N8C and M14N9) overexpressing MCF10A cells detected by transmission microscopy after 9 days of culture in matrigel without EGF. **b** The area and perimeter of organoids were quantified as described in Materials and Methods. Error bars indicate standard error of the mean for triplicate wells (*n* = 2). Asterisks indicate statistical significance with *P < 0.05; **P < 0.01.

To study whether MYB proteins promote cell cycle progression, we cultured the transduced cells in the presence or absence of EGF for 48 h following staining with propidium iodide. As expected, flow cytometric analysis showed a major change in cell cycle profiles in the absence of EGF, with a larger fraction of cells residing in the G1 phase of the cell cycle. However, under the same conditions, MYB and MYB-NFIB overexpressing cells were more abundant in the S-phase with less cells residing in G1 compared with the control cells (Fig. 1c). These results show that MYB oncoproteins promote cell cycle progression also in glandular epithelial cells. MYB and MYB-NFIB overexpressing MCF10A cells also formed significantly larger (MYB: *P* = 0.009; M14N8C: *P* = 0.0057; M14N9: *P* = 0.0193) organoids when grown in the absence of EGF in three-dimensional cultures (Fig. 2a, b). Next, we investigated whether MYB and MYB-NFIB overexpressing MCF10A cells were tumorigenic by injecting them into the flank of immunodeficient mice. No tumors were detected after a latency period of 5 months, indicating that stable MYB or MYB-NFIB overexpression is not sufficient for tumor formation of MCF10A cells (data not shown).

### MYB and MYB-NFIB activate overlapping gene sets involved in cell cycle regulation, DNA replication, and DNA repair

**Fig. 3:**
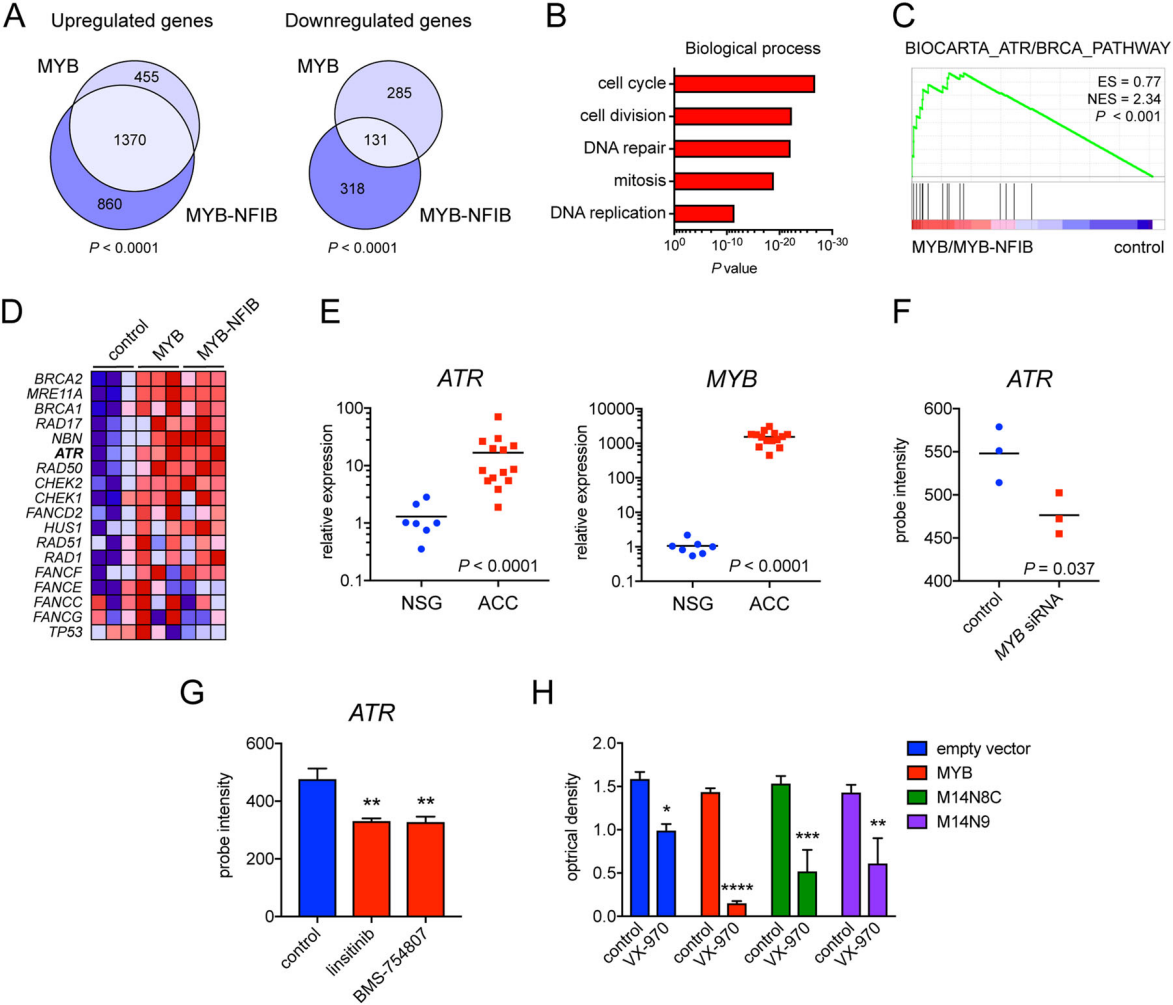
Global gene expression analysis of MYB and MYB-NFIB overexpressing human breast epithelial cells identifies activation of the ATR pathway. **a** Euler diagrams illustrating the overlap between up- and downregulated genes in MYB and MYB-NFIB overexpressing MCF10A cells compared with cells transduced with empty vectors. **b** Gene ontology analysis of genes upregulated by both MYB and MYB-NFIB in (**a**). **c** Gene set enrichment analysis of genes upregulated by both MYB and MYB-NFIB showing enrichment of the ATR/BRCA pathway. **d** Heatmap visualizing ATR pathway genes in MYB and MYB-NFIB overexpressing MCF10A cells. **e** Quantitative real-time PCR analysis of *ATR* and *MYB* expression in 14 primary ACC patient samples vs 7 normal salivary gland (NSG) tissue samples. **f** Microarray gene expression analysis of *ATR* in cultured primary ACC cells transfected with *MYB* siRNAs for 48 h. **g** Microarray gene expression analysis of *ATR* in cultured primary ACC cells treated with two different IGF1R inhibitors for 24 h. **h** Analysis of proliferation of MYB and MYB-NFIB overexpressing MCF10A cells treated with the ATR kinase inhibitor VX-970 for 24 h. Error bars indicate standard error of the mean for triplicate wells (*n* = 3). Asterisks indicate statistical significance with *P < 0.05; **P < 0.01; ***P < 0.001; ****P < 0.0001. CT – cycle threshold.

To identify target genes that promote increased proliferation of MYB and MYB-NFIB overexpressing MCF10A cells, we performed global gene expression analysis. Interestingly, there were four times more up- than downregulated genes in MYB or MYB-NFIB transduced cells, indicating that MYB is mainly a transcriptional activator. More than 50% of the upregulated genes (1370 genes) were shared between MYB and MYB-NFIB whereas only 18% (131 genes) of the downregulated genes were shared between the two MYB variants (Fig. 3a). To confirm that our models are representative of MYB-induced gene expression in ACC, we investigated the association between gene sets derived from MYB/MYB-NFIB overexpressing MCF10A cells compared with a previously published gene expression data set^16^ of MYB-positive ACC patient samples (Supplementary Fig. 2). There was a significant overlap (*P* < 0.0001) between genes upregulated by MYB/MYB-NFIB in MCF10A cells and those upregulated in MYB-positive ACC patient samples.

Next, gene ontology analysis showed that the main biological processes regulated by MYB and MYB-NFIB in MCF10A cells were those involved in cell cycle, cell division, DNA repair, mitosis, and DNA replication (Fig. 3b). Some of the activated genes are known MYB targets (for example *BIRC3*, *CDC2*, and *CXCR4*) but most of the genes have previously not been associated with MYB (Supplementary Table 1). To validate these results we performed a multiplex analysis with a qPCR panel consisting of 27 genes upregulated by MYB or MYB-NFIB. The expression of all 27 genes was increased in MYB or MYB-NFIB expressing cells compared with control cells, thus confirming the global gene expression data (Supplementary Fig. 3).

### The ATR kinase is a downstream target of MYB and MYB-NFIB

Gene Set Enrichment Analysis (GSEA) revealed that ATR/BRCA was the top activated pathway in MYB and MYB-NFIB overexpressing cells (Fig. 3c, d). Particularly, the *ATR* gene was significantly upregulated in these cells (Fig. 3d, Supplementary Table 1) as well as in 14 *MYB*-positive, ACC patient specimens (qPCR) and in eight ACC PDX models (RNA-seq) compared with normal salivary gland tissues (Fig. 3e, Supplementary Fig. 4). Analyses of publicly available gene expression data sets confirmed co-expression of *MYB* and *ATR* also in AML, adult T-ALL, and colon carcinomas and adenomas (Supplementary Fig. 5). To investigate whether *ATR* is a downstream target of MYB-NFIB in ACC, we analyzed global gene expression data sets with siRNA knockdown of MYB-NFIB in cultured ACC cells^16^. Treatment with *MYB* siRNA caused a significant decrease in the expression of *ATR*, suggesting that MYB-NFIB regulates *ATR* expression in ACC cells (Fig. 3f). Moreover, treatment of ACC cells with the IGF1R inhibitors linsitinib or BMS-754807, both known to downregulate *MYB-NFIB* expression^16^, also caused a significant decrease in the expression of *ATR* (Fig. 3g). Online chromatin immunoprecipitation data sets further indicated that MYB binds the *ATR* promoter in human and mouse cells (Supplementary Fig. 6). Taken together, these findings strongly indicate that *ATR* is a downstream target of MYB.

To assess the biological significance of ATR in cells with MYB or MYB-NFIB overexpression, we treated transduced MCF10A cells with the ATR inhibitor VX-970. The proliferation of these cells was significantly decreased by VX-970 and they also showed an increased sensitivity to ATR inhibition compared with control cells (Fig. 3h).

### Treatment with the ATR inhibitor VX-970 induces apoptosis in cultured ACC cells and inhibits growth of ACC PDXs

**Fig. 4:**
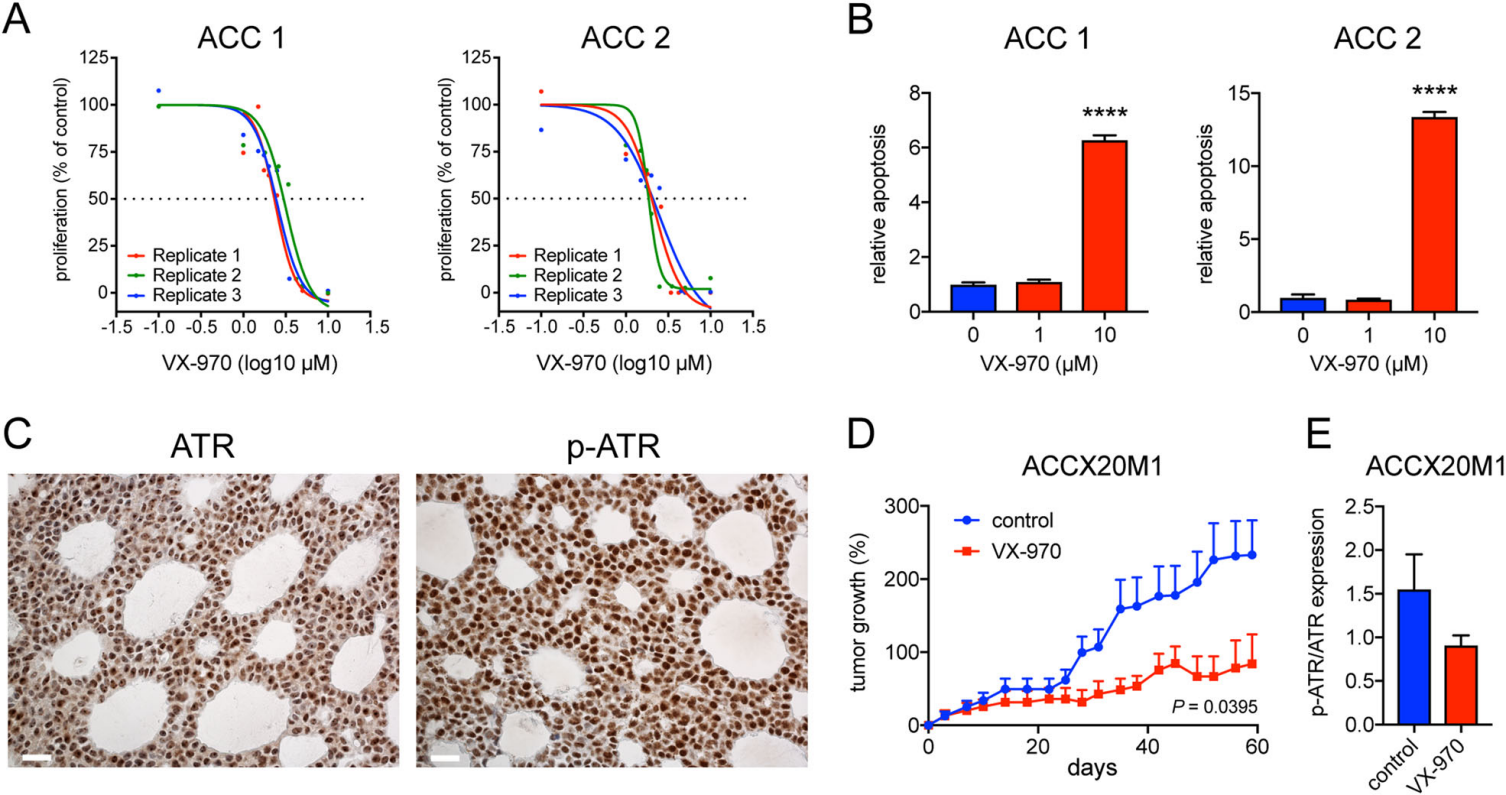
The ATR kinase inhibitor VX-970 inhibits ACC cell proliferation in vitro and ACC PDX tumor growth in vivo. **a** Dose-response curves for proliferation of short-term cultured ACC cells from two cases treated with the ATR kinase inhibitor VX-970 for 72 h. **b** Analysis of apoptosis in short-term cultured cells from two ACCs treated with VX-970 for 24 h. **c** Immunohistochemical analysis of ATR and phospho-ATR (p-ATR) expression in tissue sections from ACCX20M1 PDX tumors. **d** Tumor growth of ACC PDXs treated with oral administration of VX-970 at 60 mg/kg four times a week during 60 days (control *n* = 10; VX-970 *n* = 7). **e** Quantification of the p-ATR to ATR protein expression ratio in the VX-970 treated ACC PDXs.

Next, we investigated whether pharmacological inhibition of ATR could be a potential therapeutic strategy for ACC. Treatment of cultured MYB-NFIB positive ACC cells (ACC 1 and ACC 2) with VX-970 generated a dose-dependent decrease in proliferation and a significant increase in apoptosis (Fig. 4a, b), indicating that ATR is essential for ACC cell viability. To study whether ACCs are sensitive to ATR inhibition in vivo, we used an ACC PDX-model (ACCX20M1) with nuclear and cytoplasmic ATR expression (Fig. 4c). Treatment with VX-970 for eight weeks resulted in a significant tumor growth inhibition (Fig. 4d). Notably, one mouse even showed tumor regression (Supplementary Fig. 7). Tumor tissues from treated mice showed a decrease in phospho-ATR compared with untreated controls, which validate the on-target effect of VX-970 (Fig. 4e).

## Discussion

Activation of the master transcriptional regulator MYB through chromosomal translocation, copy number gain, or enhancer hijacking is the genomic hallmark of ACC^9,10^. Thus, MYB and its downstream oncogenic effectors are putative therapeutic targets in this disease. Here, we for the first time show that overexpression of MYB and MYB-NFIB fusions have analogous cellular consequences for cell proliferation and transcriptional regulation of downstream target genes. We also identify the DNA-damage sensor kinase ATR as a MYB and MYB-NFIB downstream effector and a potential therapeutic target in ACC.

ATR is a critical component of the cellular DNA-damage response (DDR) and is activated by replication stress (RS)^9,20^. Oncogene-induced RS promotes an active DDR that leads to induction of apoptosis or senescence, which provide natural barriers against tumorigenesis^20^. In tumors with dysfunctional DDR, oncogene-induced RS instead results in genomic instability and tumor progression^21^. In the present study, we show that MYB and MYB-NFIB activate a significant number of DNA repair genes, in addition to genes involved in cell cycle control and DNA replication. These results are in accordance with data from previously published siRNA knockdown studies of *MYB-NFIB* in ACC cells^16^. It is thus likely that overexpression of MYB and MYB-NFIB leads to an oncogene-induced RS that initiates a DDR. ACCs are inherently resistant to genotoxic stress^3,4^ and our data implies that MYB-induced upregulation of DNA repair pathways may, at least partly, be responsible for this property.

Here, we present experimental evidence that the ATR pathway is activated by MYB/MYB-NFIB in ACC through transcriptional regulation. *ATR* was significantly upregulated in non-tumorigenic breast epithelial cells overexpressing MYB or MYB-NFIB fusions as well as in MYB-positive, ACC patient samples and PDXs. Moreover, knockdown of MYB-NFIB in cultured ACC cells caused a significant decrease in *ATR* expression and there is evidence that MYB binds the *ATR* promoter in both human and mouse cells. Notably, analysis of global gene expression data sets revealed co-regulation of *MYB* and *ATR* also in other human malignancies such as for example AML, adult T-ALL, and colon carcinoma, indicating that the link between MYB and ATR is not restricted to ACC (Supplementary Fig. 5). Since cells undergoing oncogene-induced RS require an intact ATR pathway to survive^20^, we hypothesized that MYB overexpressing cells would be sensitive to ATR inhibition. Indeed, when we treated MCF10A cells overexpressing MYB or MYB-NFIB with the phase 2 ATR kinase inhibitor VX-970 these cells showed a marked decrease in cell proliferation compared with control cells. In addition, VX-970 induced a dose-dependent decrease in proliferation of cultured MYB-positive ACC cells and a significant induction of apoptosis in these cells. Treatment with VX-970 also led to significant tumor growth inhibition in ACC PDXs and even to tumor regression in one mouse. Interestingly, the PDX tumors showed both nuclear and cytoplasmic localization of ATR (Fig. 4c). This might suggest that a major function of ATR in ACC is to promote tumor cell survival, in agreement with a recent study showing a direct anti-apoptotic function of cytoplasmic ATR at the level of mitochondria^22^.

Preclinical studies have shown that ATR is essential for survival of tumor cells with defective cell cycle checkpoints or DNA repair^20^. Thus, ATR has emerged as an interesting therapeutic target and there are currently close to 40 ongoing clinical trials with ATR inhibitors in patients with various human malignancies^21^ (clinicaltrials.gov). Unfortunately, there are no available biomarkers that can identify patients who will benefit from ATR inhibition. However, the present data indicate that co-expression of *MYB* and *ATR* could be a biomarker for sensitivity to ATR inhibition. As a proof of principle, we demonstrate that ATR inhibition leads to apoptosis in MYB-positive ACC cells in vitro and growth inhibition of ACC PDX tumors in vivo. These promising results will serve as the basis for continued preclinical and clinical studies of ATR inhibitors in neoplasms with co-expression of MYB and ATR.

Depending on the mechanism of activation of *MYB* in ACC (e.g., gene fusion, copy number gain or enhancer hijacking) different isoforms of MYB are generated. We now show that the effects on cell proliferation and transcriptional regulation of downstream target genes are very similar irrespective of whether wild-type MYB proteins or MYB-fusion proteins are overexpressed. This is in line with a previous study showing similar gene expression signatures in wild-type MYB and MYB fusion-positive ACCs^23^. Furthermore, data from our laboratory suggests that there are no major clinical differences between ACCs with different genomic rearrangements of *MYB* (Persson et al., unpublished results). Taken together, these results indicate that wild-type and truncated MYB-fusion proteins have very similar oncogenic effects in glandular epithelial cells.

We also studied the transforming potential of the different MYB isoforms. MYB proteins promoted cell cycle progression and formed significantly larger organoids in three-dimensional cultures compared with controls. However, when injected into the flank of immunodeficient mice no tumors were detected after a latency period of 5 months, indicating that stable MYB or MYB-NFIB overexpression is not sufficient for tumor formation of MCF10A cells. Additional genetic alterations, such as for example mutations in the NOTCH or FGF-IGF-PI3K pathways or copy number losses involving 1p and/or 6q, are likely to be required for tumor formation^9,14,15^.

Preclinical studies of ACC suffer from the lack of established ACC cell lines and the pronounced difficulties to culture ACC cells, thus limiting the number of tumors available for experimental studies. Another limitation of this study is that only one ACC PDX-model was tested and that ATR inhibition did not result in tumor regression in more than one mouse. This is, however, in line with previous clinical observations that ACC is a treatment-resistant cancer^4^. Notwithstanding these limitations, our study has identified a new molecular target that could be exploited for further therapeutic opportunities in ACC patients.

In summary, we have shown that the DNA-damage sensor kinase ATR is a MYB and MYB-NFIB downstream effector and a novel therapeutic target in ACC. To our knowledge, this is the first time a MYB downstream gene has been successfully targeted in ACC. We also demonstrate that overexpression of MYB and MYB-NFIB fusions have analogous cellular and molecular consequences in glandular epithelial cells. Our studies thus identify a novel link between MYB and ATR in ACC that may have implications also for other types of neoplasms with activation of the *MYB* oncogene.

## Materials and methods

### Tumor and normal tissues

Fresh tumor tissue from 14 head and neck ACCs and 7 normal salivary gland (NSG) tissues were obtained from patient surgical specimens and total RNA was isolated as previously described^6,9,16^. The study was approved by the regional ethics committee in Gothenburg, Sweden (D-no: 178-08). The ethical committee waived the requirement for informed consent due to no or minimal risk for the patients and the use of patient material stripped of direct subject identifiers.

### Cell culture

MCF10A, a non-tumorigenic epithelial cell line responsive to epidermal growth factor (EGF), was purchased from ATCC. Cells were grown in DMEM/F12 medium (Invitrogen), supplemented with 20 ng/ml of human epidermal growth factor (hEGF), 0.5 μg/ml hydrocortisone, 100 ng/ml cholera toxin, 10 μg/ml insulin (Sigma-Aldrich), 5% horse serum (Gibco/Thermofisher), and 100 U/mL penicillin-streptomycin (Gibco, ThermoFisher Scientific), according to previously described culture conditions^24^. Phoenix-AMPHO and HEK293FT were grown in DMEM medium, supplemented with 10% FBS and 100 U/mL penicillin-streptomycin (Gibco, ThermoFisher Scientific). Human, MYB-NFIB fusion-positive ACC cells were cultured as previously described^6,16^. The ACC and MCF10A cells tested negative for mycoplasma prior to experiments.

### Plasmid construction and retroviral infection

Full-length coding sequences of *MYB* and two *MYB-NFIB* fusion variants linking *MYB* exon 14 to *NFIB* exons 8C (M14N8C) or 9 (M14N9) were cloned into the pMSCV vector using the *XhoI* site. Correct insertion of cDNAs was confirmed by Sanger sequencing. For retroviral infections, Phoenix-AMPHO cells were seeded in 10 cm dishes. The following day, cells were co-transfected using Lipofectamine 2000 reagent (Thermo Fisher) with 3.75 μg pPAX2 (gag-pol expressor), 1.5 μg pMDG.2 (VSV-G expressor), and 5 μg of empty pMSCV, *MYB*, or *MYB-NFIB* (M14N8C and M14N9) vectors and incubated with the transfection mix overnight at 32 °C. The day after, the transfection mix was replaced by medium specific for MCF10A cells. Fourty-eight hours after transfections, the medium was harvested, centrifuged at 2000 rpm for 30 min and filtered. The medium containing the virus particles was supplemented with polybrene (8 μg/ml) and used to infect MCF10A cells. Twenty-four hours after infections, the medium was replaced with fresh medium and 48 h later G418 (800 μg/ml) was added for clonal selection.

MCF10A cells transduced with control, *MYB* or *MYB-NFIB* (M14N8C and M14N9) retroviral vectors, were injected (2 × 10^6^ cells) into the flanks of female NOD SCID mice (*n* = 4 per group). Mice were observed for 5 months before being sacrificed and analyzed.

### Preparation of protein extracts and western blot analysis

Cells were harvested, centrifuged at 1000 rpm for 5 min, washed with phosphate-buffered saline (PBS), and cell pellets were resuspended in RIPA buffer (50 mM Tris-HCl Ph 7.5, 150 mM NaCl, 0.5% sodium deoxycholate, 1% IGEPAL® CA-630, 0.1% sodium dodecyl sulfate, 1 mM phenylmethanesulfonyl fluoride, cOmplete™ Protease Inhibitor Cocktail (all reagents supplied by Sigma-Aldrich). After 20 min incubation on ice, lysates were centrifuged at 13,000 rpm for 20 min to remove cell debris. Protein extracts were quantified using the Pierce™ BCA Protein Assay Kit (Thermo Fisher Scientific), according to manufacturer instructions. Protein extracts were loaded on 8% gels (SureCast™ Acrylamide Solution (40%), Invitrogen) and transferred to PVDF membranes (Fisher Scientific). Western blot analyses were performed using the following primary antibodies: c-MYB (sc-74512, Santa Cruz Biotechnology) and Actin (sc-1616, Santa Cruz Biotechnology). HRP-conjugated secondary antibodies used were anti-mouse IgG (NXA931, GE Healthcare, Fisher Scientific) and anti-rabbit IgG (NA934, GE Healthcare, Fisher Scientific). Antibody detection was performed with enhanced chemiluminescence (ECL) (Thermo Fisher Scientific).

### Proliferation and apoptosis assays

One thousand MCF10A cells were seeded per well in 96-well plates and metabolic activity was quantified using the CellTiter 96^®^ AQueous MTS Reagent kit (Promega), following manufacturer instructions. Four thousand ACC cells were seeded per well in black 96-well plates (BD) and treated the next day with different concentrations of the ATR kinase inhibitor VX-970 (also known as VE-822 or M6620; Selleck Chemicals) or DMSO as control. Cells were assayed 72 h later with the Alamar blue reagent (Thermo Fisher Scientific) according to instructions of the manufacturer. For apoptosis assays, 8000 ACC cells were seeded in white-walled 96-well plates (BD) and treated the next day with 1 or 10 μM VX-970 for 24 h. Apoptosis was assayed with the Caspase-Glo 3/7 reagent (Promega).

### Cell cycle analysis

Three million MCF10A cells were seeded in T75 flasks and cultured for 48 h with or without EGF. Cells were harvested, washed with PBS, fixed in 70% ethanol and kept at −20 °C overnight. The following day, cells were washed twice with PBS to remove ethanol, and resuspended in 1 ml of PBS containing RNaseA (100 μg/ml) and propidium Iodide (50 μg/ml) (Sigma-Aldrich). Cell cycle analysis was performed using a NovoCyte Cytometer using the NovoExpress 1.2.1 software.

### RNA isolation, cDNA synthesis, and quantitative real-time PCR (qPCR)

Total cellular RNA was extracted from MCF10A cells using the TRIzol Reagent (Thermo Fisher Scientific), and from short-term cultured ACC cells using the RNeasy Micro Kit (Qiagen), following the manufacturers’ instructions. TRIzol extracted RNA was incubated with deoxyribonuclease I (DNase I, Amp Grade; Invitrogen) at 37 °C for 1 h and reverse transcribed using a high capacity RNA-to-cDNA kit. RNeasy extracted RNA was reverse transcribed with the iScript cDNA Synthesis Kit (Bio-Rad). Expression of *MYB* and 27 MYB target genes were analyzed using custom TaqMan array plates (Supplementary Table 2) and with single TaqMan assays (Thermo Fisher Scientific) for *ATR* (Hs00354787_m1) and *MYB* (Hs00920556_m1*). *UBC* (Hs01871556_s1) was used as a reference gene.

### Three-dimensional cell cultures

Three-dimensional MCF10a cell cultures were established as previously described^25^. In brief, cells were embedded in growth factor reduced Matrigel (Corning™ Matrigel™) and seeded on Matrigel precoated slides (Merck™ Millicell™ EZ Slides, Millipore). The embedded cells were fed with medium without EGF every second day. Organoids (cell acini) were fixed with 4% paraformaldehyde for 10 min and washed twice with PBS. Area and perimeter of the organoids were estimated using the ImageJ software.

### ACC PDX drug testing

The antitumor activity of the ATR kinase inhibitor VX-970 (LC Laboratories) was studied in the ACC PDX-model, ACCX20M1^26^, at South Texas Accelerated Research Therapeutics (START) under Institutional Animal Care and Use Committee-approved guidelines. Six to eight weeks old athymic nude mice (Charles River Labs) were implanted with ACC tumor fragments. Once tumors reached 150–250 mm^3^, animals were randomized to receive control (no treatment) or VX-970 administered orally at 60 mg/kg once a day, four days a week, until study endpoint (60 days). Animals were observed daily and tumor volume and animal weight were measured twice a week using a digital caliper and scale. Tumor dimensions were converted to volume using the formula: TV (mm^3^) = width^2^ (mm) × length (mm) × 0.52. Percent tumor growth inhibition values were calculated and reported for treatment group versus control using initial and final tumor measurements.

### Analysis of ATR expression in PDX tumor tissues

Proteins from frozen ACC PDX tumors were isolated with the TissueLyzer II (QIAGEN, 85300) using 1.5 mm beads. 120 μl RIPA buffer (50 mM Tris-HCl pH 7.6, 150 mM NaCl, 1% v/v NP-40, 0.5% w/v sodium deoxycholate, 0.1% w/v SDS) plus protease (Roche, 4693159001), and phosphatase (Sigma, P5726) inhibitors were added to tumor samples, which were lysed at 30 Hz for 40 s. The homogenized tissues were centrifuged at 12,000 × *g* for 5 min at 4 °C and the protein extracts were stored at −80 °C in Laemmli buffer to a final concentration of 1X. For Western Blot analysis, 20 μg of proteins were loaded in 8% acrylamide gels, separated by electrophoresis and transferred onto nitrocellulose membranes for 2 h at 4 °C. The membranes were blocked with 5% milk in TBS containing 0.1% Tween-20 (TBS-T) and incubated overnight at 4 °C with primary antibodies against total ATR (Cell Signaling, 2790) or phospho-ATR (Thr1989) (GeneTex, 128145) diluted 1:1000 in 5% BSA in TBS-T. After washing three times for 5 min with TBS-T, the membranes were incubated with an anti-rabbit IgG-HRP antibody (Santa Cruz, sc-2313) diluted 1:10,000 in 5% BSA in TBS-T for 1 h at RT. Quantification of the Western blots was carried out using ImageJ software.

### RNA-sequencing (RNA-seq)

Ten ACC PDX tumors and six normal human salivary gland tissues were collected in RNAlater (ThermoFisher) and processed using TruSeq Stranded mRNA Library Kit (Illumina). RNA extracted from these samples was sequenced on an Illumina NovaSeq 6000 at a depth of 50 M reads. Gene expression levels were determined from RNA-seq after trimming reads for Illumina adapters with Trimmomatic 0.36, aligning reads to the hg19 genome with TopHat 2.1.1, and assigning reads to exons from the TxDb.Hsapiens.UCSC.hg19.knownGene R package with R 3.5.1. Differential gene expression between tumor and normal salivary gland samples was calculated with DESeq2 v1.20.0.

### Global gene expression analysis

Biotin allonamide triphosphate-labeled cRNA from transduced MCF10A cells was synthesized from total RNA and hybridized to Human Gene 1.0 ST gene chips (Affymetrix, Santa Clara, CA) as recommended by the manufacturer. Probe summarization and quantile normalization were carried out by Robust multiarray analysis (RMA) using the Expression Console Software v1.1.2 (Affymetrix). The microarray data are available from the Gene Expression Omnibus (GEO) database (Accession No. GSE136095). Expression of *ATR* in cultured *MYB-NFIB* positive ACC cells treated for 48 h with *MYB* siRNAs and from serum-starved ACC cells treated for 24 h with IGF1R inhibitors (linsitinib or BMS-754807) and insulin (5 μg/ml) was analyzed from previously published microarray data Accession No. GSE76094^16^. Co-expression of *MYB* and *ATR* in acute myeloid leukemia (AML), adult T-cell acute lymphoblastic leukemia (T-ALL), and colon carcinomas and adenomas was analyzed with the publicly available R2 Genomics Platform (http://r2.amc.nl).

### Immunohistochemistry

Formalin-fixed, paraffin-embedded tissue sections from tumors of the ACC PDX-model ACCX20M1 were deparaffinized, and antigen epitopes were retrieved with EnVision FLEX Target Retrieval Solution pH 9 (Agilent). Slides were rinsed and endogenous peroxidase activity was blocked with the EnVision Flex Mini Kit (Agilent). Slides were incubated overnight at 4 °C with antibodies to ATR (GTX128146) or p-ATR (S428, Abcam). Bound antibodies were detected with HRP-conjugated secondary antibodies and visualized with the EnVision FLEX DAB + Chromogen substrate. Control sections were treated identically but without primary antibodies. Stained sections were photographed with an Olympus BX45 microscope (Tokyo, Japan) equipped with a Nikon Digital Sight DS-U2 camera (Tokyo, Japan) and visualized with the NIS-Elements F 2.30 software.

### Statistical analysis

One-way ANOVA or independent samples *t*-tests were used to estimate significant differences between groups with post hoc *F* tests to confirm the assumption of equal variance. All statistical tests were two-sided. A *P* value of <0.05 was considered statistically significant. Non-linear regression of dose-response experiments was carried out in Prism 7 (Graphpad Software). Microarray data was analyzed for differentially expressed genes and gene ontologies with Nexus Expression 3.0 (BioDiscovery) using the Benjamini–Hochberg method and false discovery rate-corrected *Q* values of <0.05. Gene Set Enrichment Analysis (GSEA) and heatmap visualization were done with GSEA v3.0 (Broad Institute). Statistically significant overlap between gene sets was estimated with Chi-square tests.

## Supplementary information

Supplementary Table 1

Supplementary Table 2

Supplemental Figure legends

Supplementary Figure 1

Supplementary Figure 2

Supplementary Figure 3

Supplementary Figure 4

Supplementary Figure 5

Supplementary Figure 6

Supplementary Figure 7
