# Supplementary material for: ATR is a MYB regulated gene and potential therapeutic target in adenoid cystic carcinoma
Source: Oncogenesis. 2020 Jan 30;9(1):5. doi: 10.1038/s41389-020-0194-3 (PMC6992744; doi:10.1038/s41389-020-0194-3)
Supplement: Supplementary file 3 — Supplemental Figure legends [file 41389_2020_194_MOESM3_ESM.docx]

**Supplementary Figure 1.** **Ectopic expression of MYB and MYB-NFIB in MCF10A cells.** Western blot analysis of MCF10A cells transduced with retroviral expression vectors with MYB or MYB-NFIB fusions (N9 = M14N9, 8C = M14N8C) or empty vector (ctrl) using a MYB antibody. Proteins extracted from K562 leukaemia cells served as positive control for MYB expression. An antibody against GAPDH was used as loading control.

**Supplementary Figure 2. Gene expression profiles of MYB-infected MCF10a cell lines overlap with those detected in ACC tumours.**Euler diagrams showing overlap between gene expression profiles of MYB and M14N9 cell lines and a previously published data set (GSE88804, https://www.ncbi.nlm.nih.gov) of 13 MYB-positive ACCs versus 7 normal salivary gland tissues

**Supplementary Figure 3. Validation of global gene expression data.** Quantitative real-time PCR analyses of 27 upregulated genes in MCF10A cells expressing MYB or a MYB-NFIB fusion (M14N9). Each colored bar indicates the expression of a selected gene in transduced MCF10A cells. The dashed line indicates the expression in cells transduced with an empty vector. Data comes from two independent experiments. Error bars indicate standard error of the mean.

**Supplementary Figure 4. *ATR* is upregulated in ACC patient-derived xenograft (PDX) models.** Expression of *ATR* and *MYB* transcripts in 10 ACC PDX models compared with six normal salivary gland controls (NGS) analysed with RNA-sequencing. Nd – not detected.

**Supplementary Figure 5. *MYB* and *ATR* are co-expressed in acute myeloid leukemia, adult T-cell acute lymphoblastic leukemia, and colon carcinomas and adenomas.** Analyses of *MYB* and *ATR* expression in benign and malignant neoplasms with the publicly available R2 Genomics Platform (http://r2.amc.nl).

**Supplementary Figure 6. MYB binds the *ATR* promoter in human and mouse cells.** Chromatin immunoprecipitation analysis showing binding of MYB to the promoter region of human and mouse *ATR* genes on chromosomes 3 and 9, respectively. Data obtained with online tools available at Cistrome Data Browser (http://dc2.cistrome.org/#/).

**Supplementary Figure 7. Treatment with the ATR kinase inhibitor VX-970 can induce tumor regression in ACC PDXs.** Oral administration of VX-970 at 60 mg/kg four times a week during 60 days lead to tumor regression in one mouse in ACCX20M1 PDXs (control n=10; VX-970 n=1).
