# Supplementary figures and images for: ATR is a MYB regulated gene and potential therapeutic target in adenoid cystic carcinoma
Source: Oncogenesis. 2020 Jan 30;9(1):5. doi: 10.1038/s41389-020-0194-3 (PMC6992744; doi:10.1038/s41389-020-0194-3)

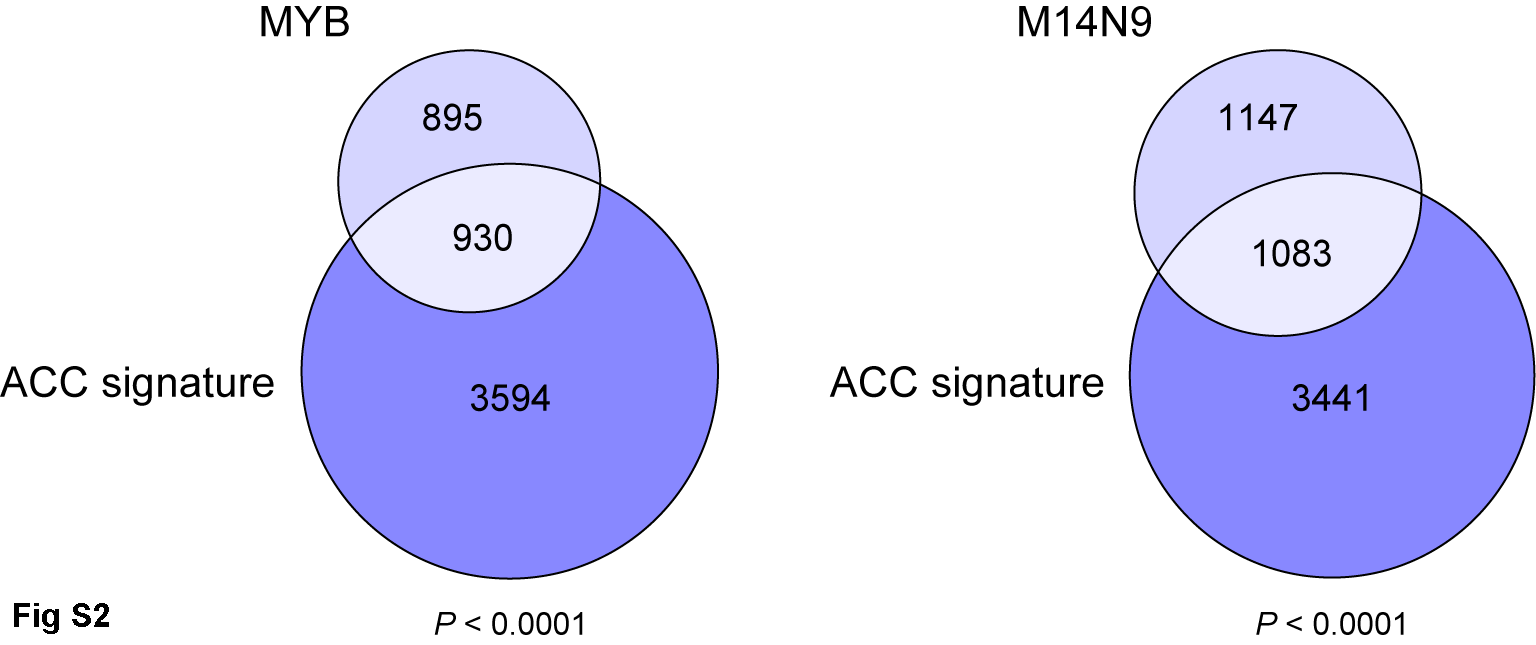

Supplement: Supplementary file 5 — Supplementary Figure 2 [file 41389_2020_194_MOESM5_ESM.tif]

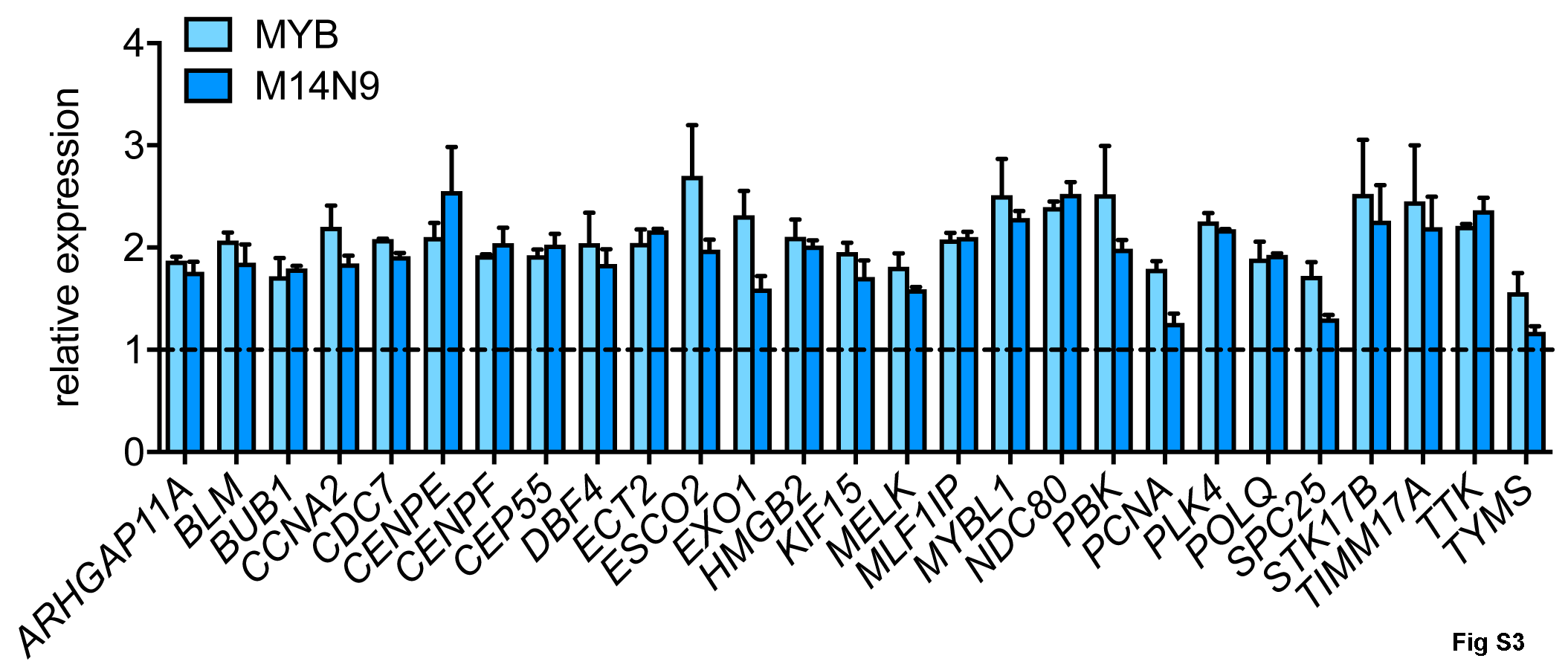

Supplement: Supplementary file 6 — Supplementary Figure 3 [file 41389_2020_194_MOESM6_ESM.tif]

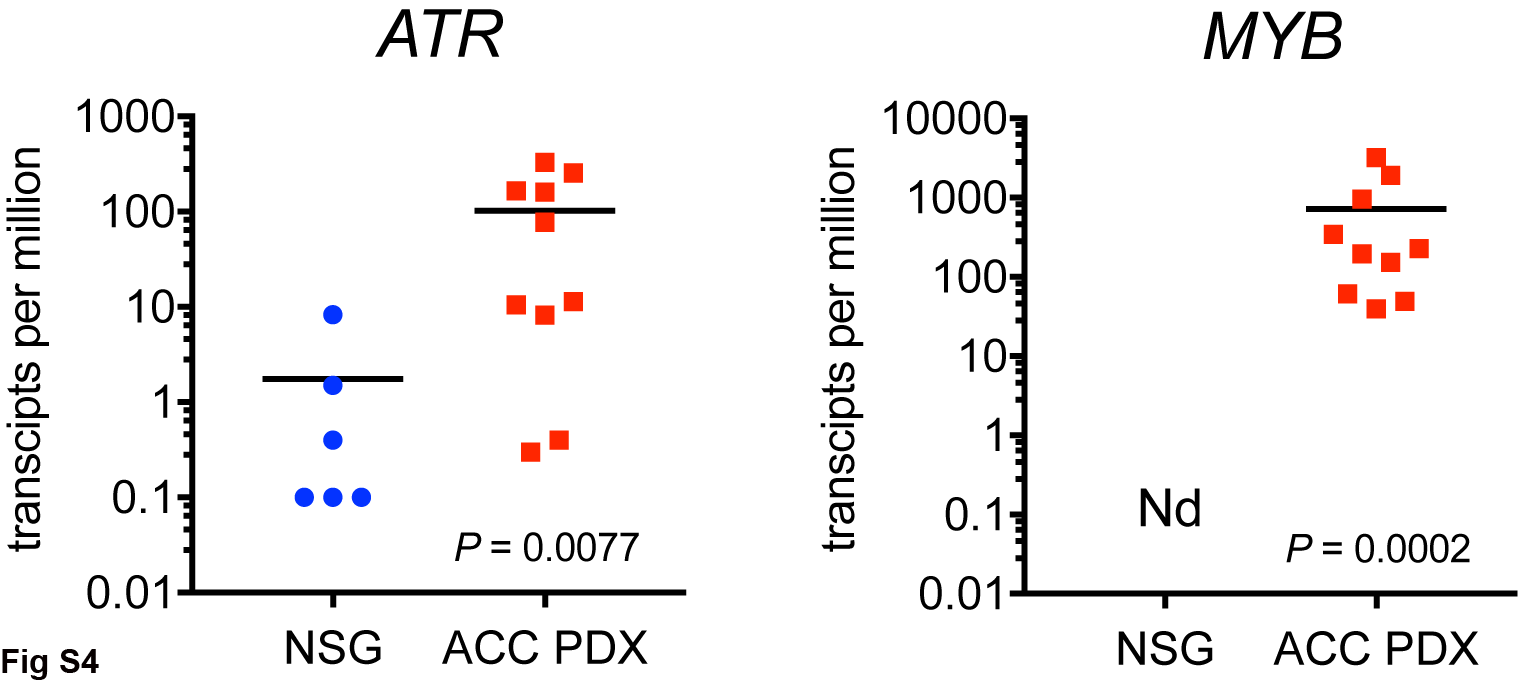

Supplement: Supplementary file 7 — Supplementary Figure 4 [file 41389_2020_194_MOESM7_ESM.tif]

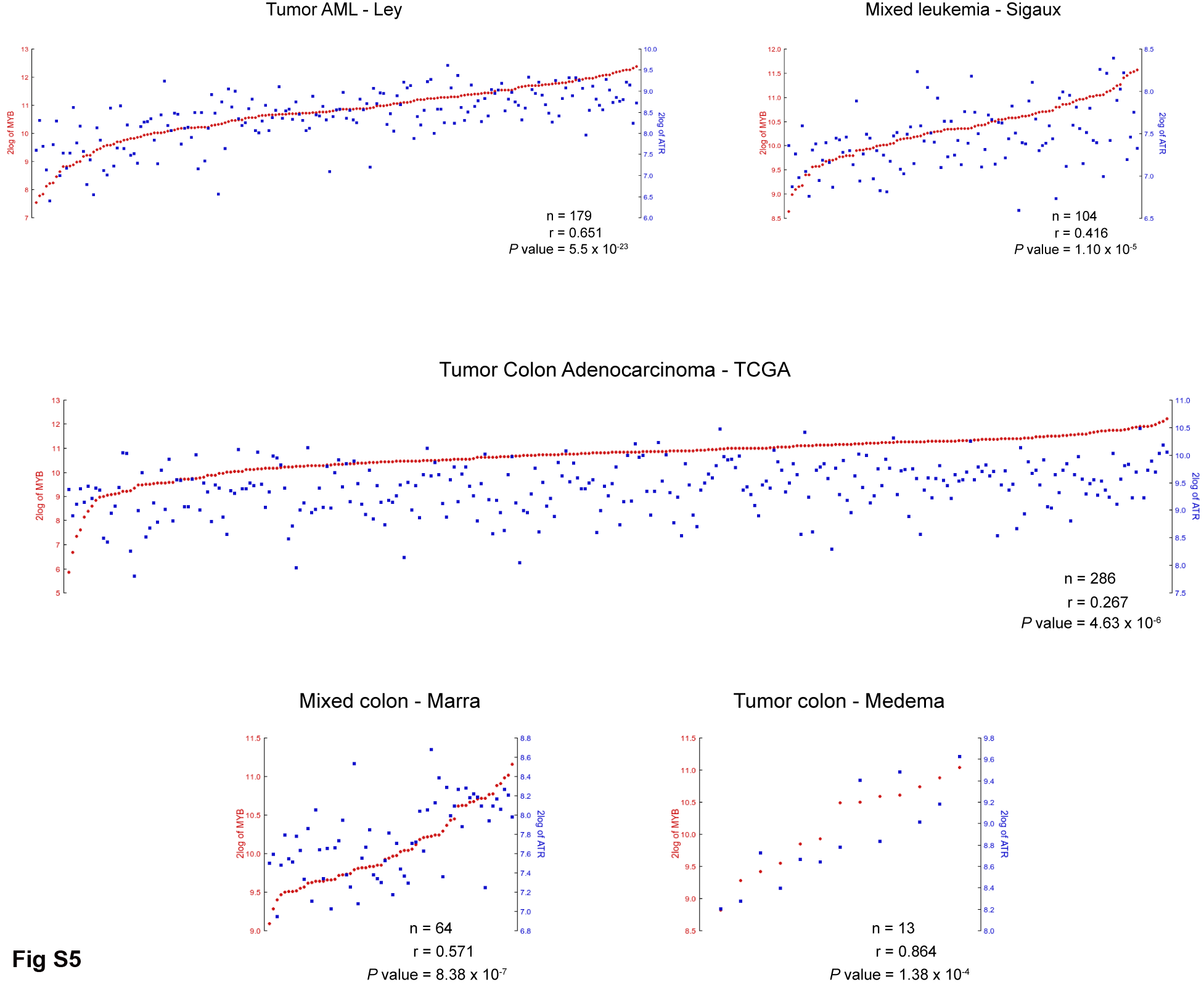

Supplement: Supplementary file 8 — Supplementary Figure 5 [file 41389_2020_194_MOESM8_ESM.tif]

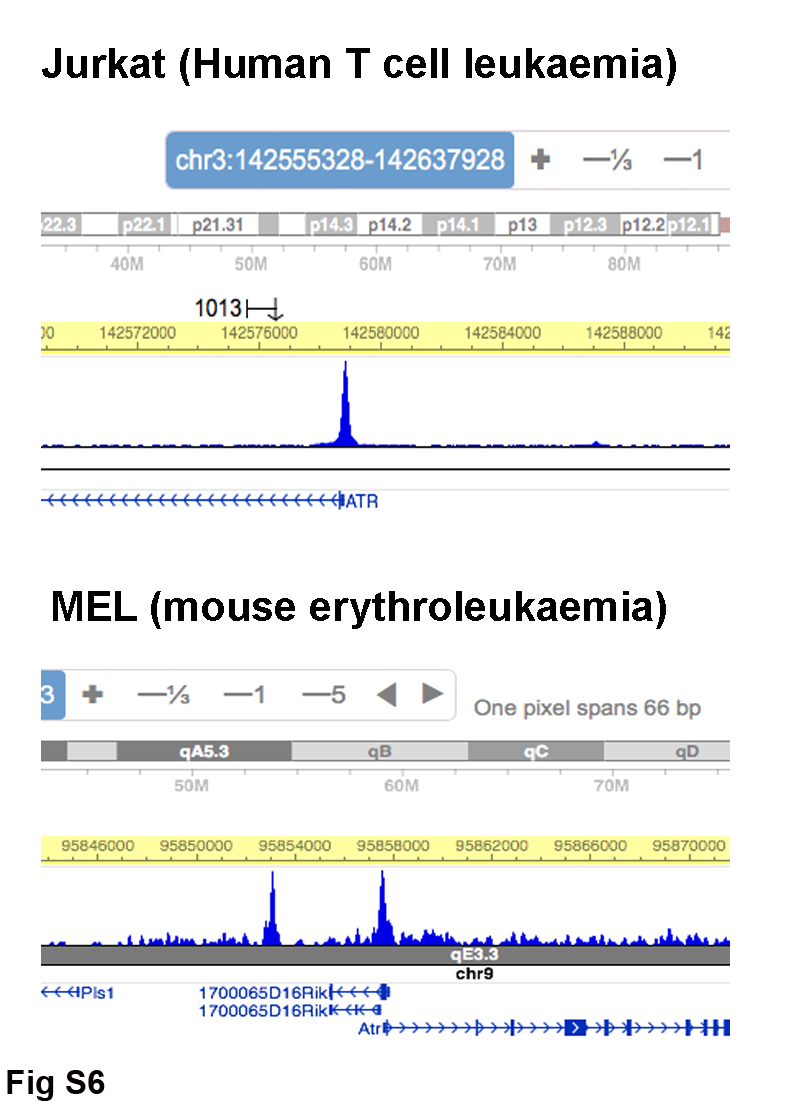

Supplement: Supplementary file 9 — Supplementary Figure 6 [file 41389_2020_194_MOESM9_ESM.tif]

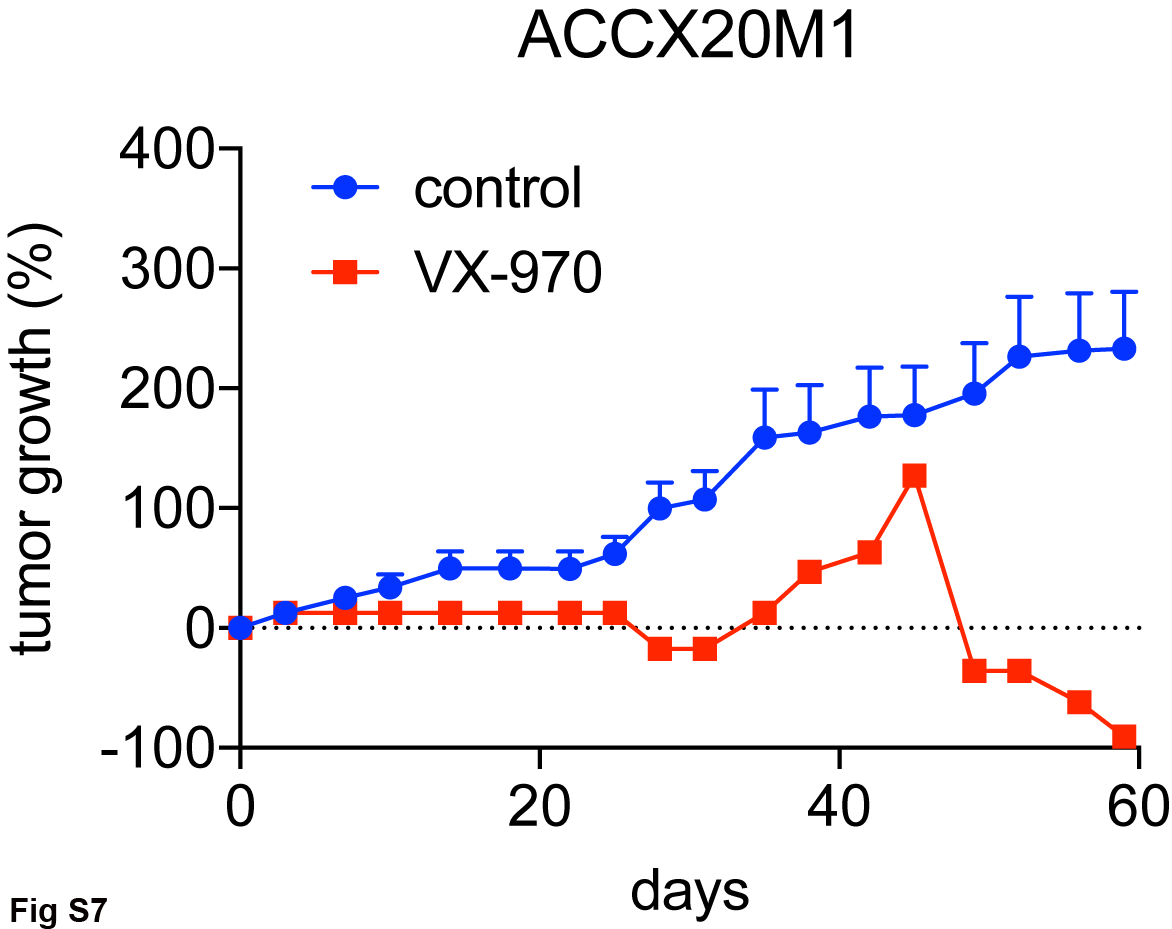

Supplement: Supplementary file 10 — Supplementary Figure 7 [file 41389_2020_194_MOESM10_ESM.tif]
